# Interplay of Staphylococcal and Host Proteases Promotes Skin Barrier Disruption in Netherton Syndrome

**DOI:** 10.1016/j.celrep.2020.02.021

**Published:** 2020-03-03

**Authors:** Michael R. Williams, Laura Cau, Yichen Wang, Drishti Kaul, James A. Sanford, Livia S. Zaramela, Shadi Khalil, Anna M. Butcher, Karsten Zengler, Alexander R. Horswill, Christopher L. Dupont, Alain Hovnanian, Richard L. Gallo

**Affiliations:** 1 Department of Dermatology, University of California, San Diego, San Diego, CA 92093, USA; 2 SILAB, R&D Department, Brive, France; 3 INSERM, UMR 1163, Laboratory of Genetic Skin Diseases, Imagine Institute and Université Paris Descartes-Sorbonne Paris Cité, Paris, France; 4 J. Craig Venter Institute, La Jolla, CA 92093, USA; 5 Department of Pediatrics, University of California, San Diego, San Diego, CA 92093, USA; 6 University of Virginia School of Medicine, Charlottesville, VA 22908, USA; 7 Center for Microbiome Innovation, University of California, San Diego, San Diego, CA 92093, USA; 8 Department of Bioengineering, University of California, San Diego, CA 92093, USA; 9 Department of Veterans Affairs Denver Health Care System, Denver, CO, USA; 10 Department of Immunology and Microbiology, University of Colorado Anschutz Medical Campus, Aurora 80045, CO, USA; 11 These authors contributed equally; 12 Lead Contact

## Abstract

Netherton syndrome (NS) is a monogenic skin disease resulting from loss of function of lymphoepithelial Kazal-type-related protease inhibitor (LEKTI-1). In this study we examine if bacteria residing on the skin are influenced by the loss of LEKTI-1 and if interaction between this human gene and resident bacteria contributes to skin disease. Shotgun sequencing of the skin microbiome demonstrates that lesional skin of NS subjects is dominated by *Staphylococcus aureus (S. aureus)* and *Staphylococcus epidermidis* (*S. epidermidis*). Isolates of either species from NS subjects are able to induce skin inflammation and barrier damage on mice. These microbes promote skin inflammation in the setting of LEKTI-1 deficiency due to excess proteolytic activity promoted by *S. aureus* phenol-soluble modulin α as well as increased bacterial proteases staphopain A and B from *S. aureus* or EcpA from *S. epidermidis*. These findings demonstrate the critical need for maintaining homeostasis of host and microbial proteases to prevent a human skin disease.

## In Brief

Williams et al. show how an abnormal skin microbiome promotes inflammation associated with Netherton syndrome, a monogenic disorder in the human protease inhibitor SPINK5. Subjects with Netherton syndrome have excess colonization by *S. aureus* or *S. epidermidis* that then produce and promote increased protease production in the epidermis.

## Graphical Abstract

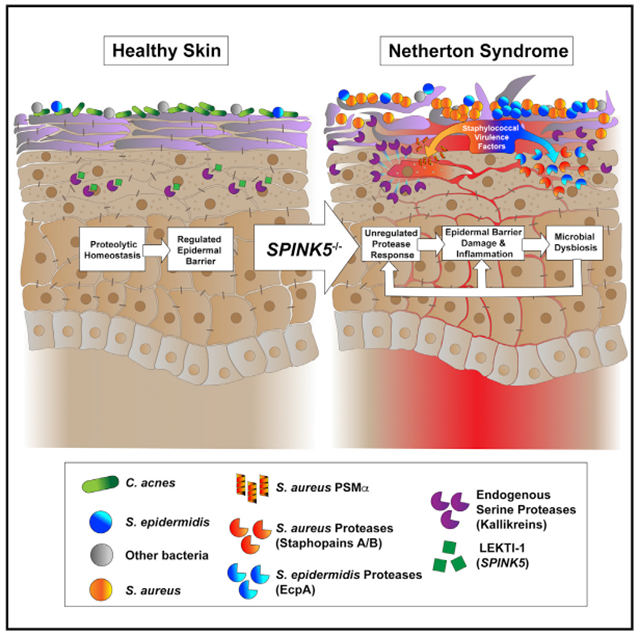

## INTRODUCTION

The identity and abundance of microbes on epithelial surfaces have been linked to important outcomes in human disorders, such as inflammatory bowel diseases, neurological disorders, obesity, cancer, diabetes, autoimmune disorders, and skin diseases (Gilbert et al., 2018; Helmink et al., 2019; Kho and Lal, 2018; Schmidt et al., 2018). Symptoms associated with microbial dysbiosis are generally thought to reflect the influence of specific members of the microbial community on health. For example, the presence of *Staphylococcus aureus (S. aureus)* on subjects with atopic dermatitis (AD) is strongly associated with disease (Alsterholm et al., 2017; Gong et al., 2006; Kong et al., 2012; Leyden et al., 1974; Paller et al., 2019). However, even in this example, *S. aureus* is not universally found on all AD subjects, and the role of the microbe in AD pathogenesis continues to be debated (Paller et al., 2019). It also remains unclear why *S. aureus* promotes inflammation in the absence of infection in AD but not in healthy individuals. Although much progress has been made in the past decade toward understanding the roles of the microbiome in human health, the inability to connect host genotypes to microbial functions has made it difficult to establish causality for microbes in human inflammatory diseases.

The contribution of the host to control the composition of the resident microbiome is poorly understood. In the case of AD, mutations in the skin barrier protein filaggrin (FLG) represent the most significant known genetic risk factor (Esparza-Gordillo et al., 2009; Morar et al., 2007). FLG mutations in AD have also been associated with an increase of *S. aureus* colonization in subjects (Clausen et al., 2017). However, not all subjects with FLG mutations have dysbiosis of the microbiome, and most AD subjects do not have FLG mutations. The complexity of a multifactorial disease such as AD makes it difficult to connect skin inflammation to microbial dysbiosis through a human genetic abnormality. Several murine models have been useful to show how host genetic modifications can influence microbial colonization or inflammation, but much work remains to establish this link in humans (Kobayashi et al., 2015; Nakatsuji et al., 2016). Overall, there is a paucity of examples of a canonical pathway connecting a human gene to microbial dysbiosis and subsequent disease symptoms.

In this study, we investigated subjects with the autosomal-recessive skin disease Netherton syndrome (NS) to understand the relationship between human genetic mutations and the role of the microbiome in skin disease. NS subjects have a single gene mutation in the serine protease inhibitor Kazal type 5 gene *(SPINK5)* that leads to a loss of function of the protein lymphoepithelial Kazal-type-related protease inhibitor (LEKTI-1) (Chavanas et al., 2000). Mechanistically, this mutation leads to an increase in epidermal serine protease activity that subsequently leads to skin barrier damage and inflammation. The mouse *Spink5* knockout is lethal shortly after birth because of a severely impaired epidermal skin barrier (Bonnart et al., 2010; Briot et al., 2009; Descargues et al., 2005). In humans, NS subjects typically have a generalized skin inflammatory phenotype at birth that reflects the abnormal development of the epidermal barrier due to the lack of LEKTI-1 activity. However, patients with NS improve with age, and adults do not show a generalized skin phenotype. Adults with NS typically have limited and distinct skin locations with epidermal breakdown, and this clinical phenotype changes over time. On the basis of this clinical finding, we investigated if the function of the local skin microbiome could contribute to the localized and transient nature of skin disease in adult NS subjects.

Recently *S. aureus* was shown to damage normal human keratinocytes through the release of bacterial proteases and the production of phenol-soluble modulin alpha (PSMα) peptides that induce human endogenous serine protease activity on the skin surface (Liu et al., 2017; Nakagawa et al., 2017; Nakatsuji et al., 2016; Williams et al., 2017, 2019). These findings showed that bacterial products can negatively modulate the host skin barrier either by direct action of their own secreted proteases or by controlling host protease responses.

On the basis of the variable clinical phenotype of adult NS subjects and previous findings with *S. aureus*, we hypothesized that the mutation of the human protease inhibitor gene *SPINK5* could enable the skin microbiome to further exacerbate the clinical phenotype of NS beyond the effects on epidermal differentiation observed in newborns. This hypothesis was further supported by the fact NS subjects can also frequently become infected by *S. aureus* (Chao et al., 2005; Renner et al., 2009; Zhvania et al., 2017). Unexpectedly, all ten NS subjects assessed in our study were dominated by *S. aureus* and/or *Staphylococcus epidermidis* (*S. epidermidis*). Furthermore, aside from the previously established capacity of *S. aureus* to alter proteolytic balance in the epidermis, we discovered that an *S. epidermidis* cysteine protease may also play an important role in damaging the skin and inducing inflammation. Overall, this study allows for a better understanding of how interactions between systems of microbial and human proteases activity are important to maintain homeostasis and contributes to disease in NS.

## RESULTS

### The Skin Microbiome of NS Is Distinct from Healthy Skin

**Figure 1.**
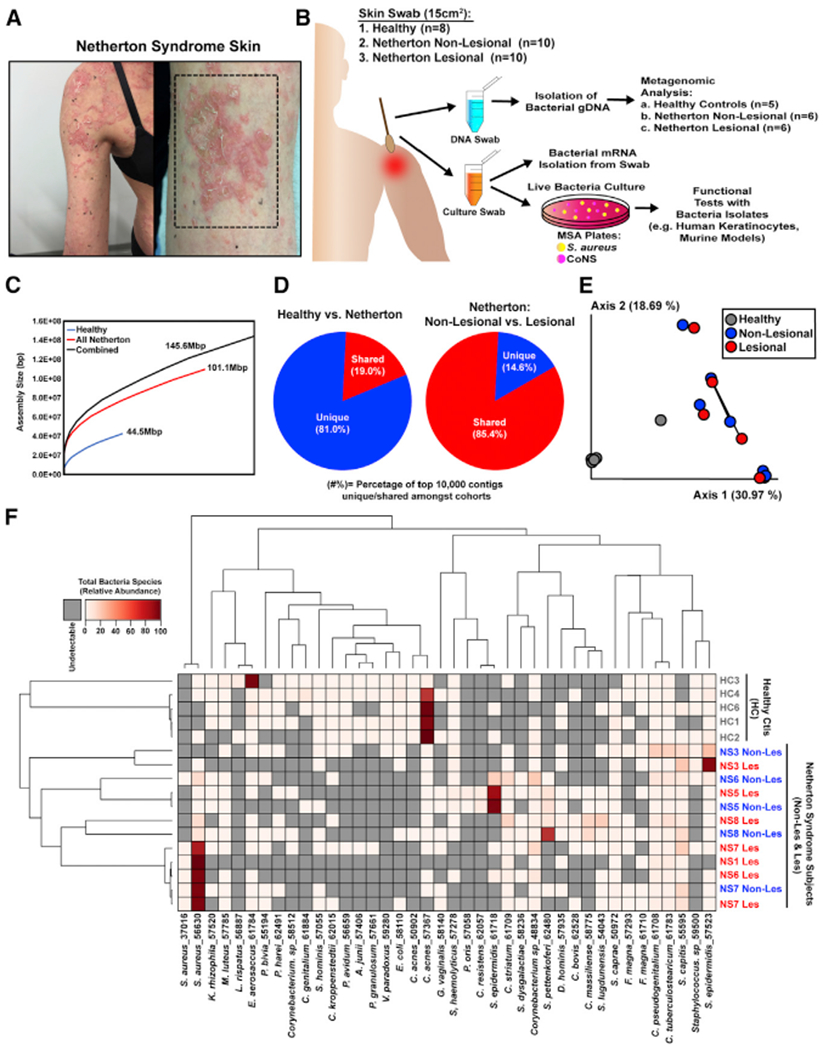
Netherton Skin Microbiome Differs from Healthy Skin (A) Representative picture of Netherton syndrome skin with severe disease. (B) Workflow of Netherton skin microbiome collection and analysis. (C) QUAST plots to assess the size of contigs for all assemblies. Three different co-assemblies were performed: reads from all samples (healthy and infected) (black), reads from only the Netherton cohort (red), and reads from only the healthy samples (blue). (D) Pie chart representing the percentage of the top 10,000 contigs unique (blue) or shared (red) between healthy subjects and Netherton syndrome patients (left chart) and between Netherton syndrome non-lesional skin and lesional skin (right chart). (E)Principal-coordinates analysis (PCoA) plot of beta diversity among samples using the Bray-Curtis dissimilarity metric. Each dot represents an individual swab. Swabs fromlesional and non-lesional skin from the same subject are connected by a black line. (F) Hierarchical clustering of samples showing the top 40 most prevalent species across all samples. See also Figures S1–S3.

Swabs were collected from the skin of ten subjects with NS whose clinical diagnosis was confirmed by the presence of causative mutations in *SPINK5*. Swabs were collected on different anatomical areas of the trunk and extremities according to the presence of a lesion. Several subjects were also screened at multiple time points. Areas sampled included the abdomen, thigh, and arm, which have similar microbial content under healthy conditions (Table S1). The number of collected samples varied from one subject to another depending of the number of visits the subject had during the time of the study. The microbiota from individual swab samples were studied by both deep shotgun sequencing and culturing of live individual bacterial isolates (Figures 1A and 1B; Figure S1). To permit adequate depth of sequencing, only samples with less than 98% of human genomic DNA contamination were fully sequenced (five healthy subjects and six NS subjects). Meta-analysis of the assembled contigs revealed major differences between the healthy and NS cohorts but a similarity between bacterial DNA sequences from lesional and non-lesional sites within the NS group (Figures 1C–1E; Figure S2). Of the 40 most abundant bacterial species and strains, *Cutibacterium acnes* predominated on healthy control subjects, except for one subject with *Enhydrobacter aerosaccus*, while both the lesional and non-lesional skin of NS subjects had increased staphylococcal species, including both *S. epidermidis* and *S. aureus* (Figure 1F; Figure S3). Overall the most abundant species on either healthy or NS skin samples was frequently greater than 30% of the entire community. These data show a large difference between the compositions of the overall bacterial community of NS subjects compared with controls. This difference in bacterial communities between healthy skin and NS exceeds prior reports of the difference in the microbiome between subjects with AD and controls (Byrd et al., 2018).

**Figure 2.**
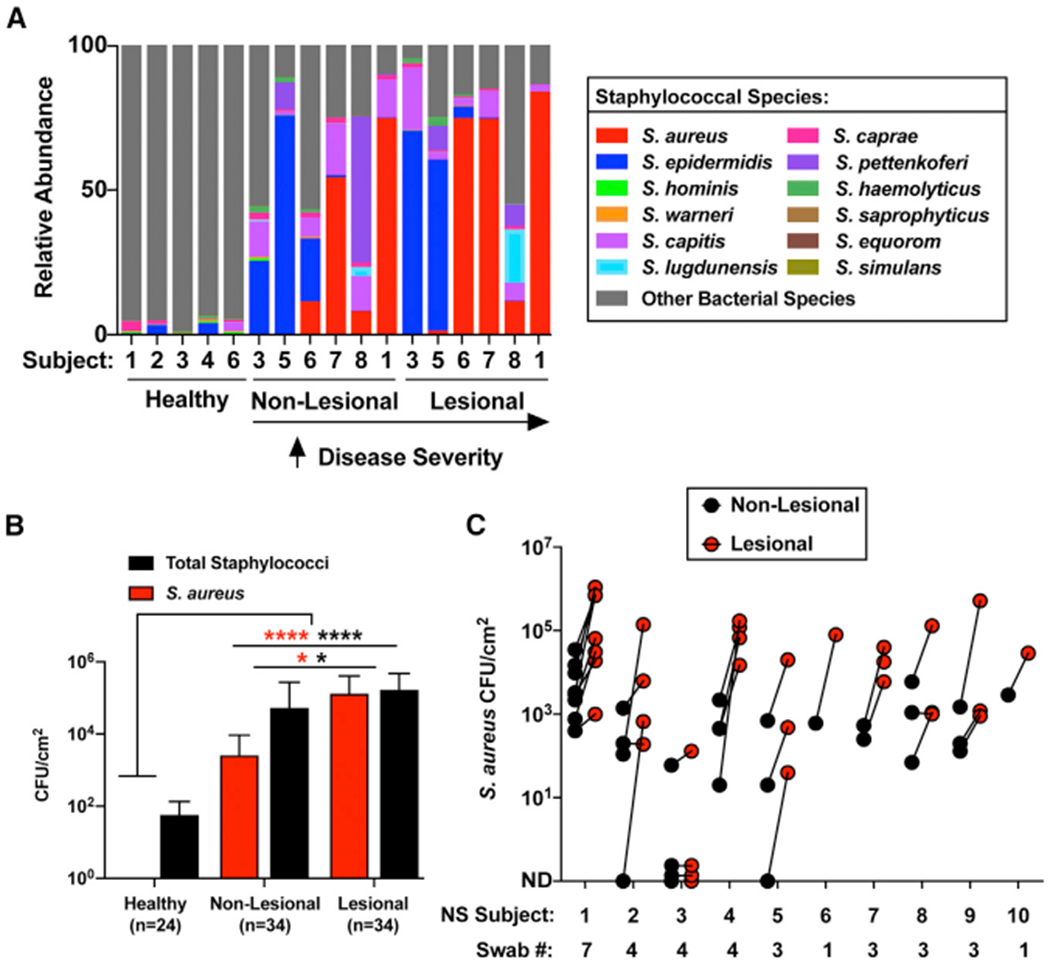
*Staphylococcus aureus* Colonization Is Increased on Netherton Syndrome Skin (A) Percentage relative abundance of staphylococcal species within the total bacterial population on healthy controls, NS non-lesional, and NS lesional skin. NS subjects are arranged according to disease severity. (B) *S. aureus* (red) and total staphylococci (black) colony-forming units (CFUs) per square centimeter of skin from healthy controls and NS non-lesional and lesional skin (n, number of swabs assessed per condition). Results represent mean ± SEM, and the non-parametric unpaired Kruskal-Wallis test was used to determine statistical significance: *p < 0.05, **p < 0.01, ***p < 0.001, and ****p < 0.0001. (C) *S. aureus* CFUs per square centimeter of skin of NS non-lesional (black) and lesional (red) skin swabs at different visits (swab number) for each subject within the NS cohort. Each dot represents a swab sample. Different numbers of swabs were collected for the different subjects depending on the number of visits they had during the time of the study. See also Figure S4.

Analysis of the diversity of staphylococcal species on NS and healthy controls showed an increase in the relative abundance of *S. aureus* and *S. epidermidis* on NS subjects regardless of whether the swabs were collected from lesional or non-lesional sites (Figure 2A; Figure S1). However, analysis of the absolute abundance of total live staphylococci and *S. aureus* on skin by colony counting showed a significant increase on the lesional skin of NS subjects compared with non-lesional skin sites and skin from healthy controls (Figure 2B). Also, all of the NS subjects in this study were colonized by *S. aureus* at least at one time point from multiple collections (Figure 2C; Figure S4). Together, these findings unexpectedly showed that *S. aureus* and *S. epidermidis* dominate NS skin in terms of both absolute abundance of live colonies and relative species abundance as determined by shotgun metagenomic analysis.

### *S. aureus* Phenol-Soluble Modulin α Promotes Epidermal Protease Activity that Is Amplified in NS

**Figure 3.**
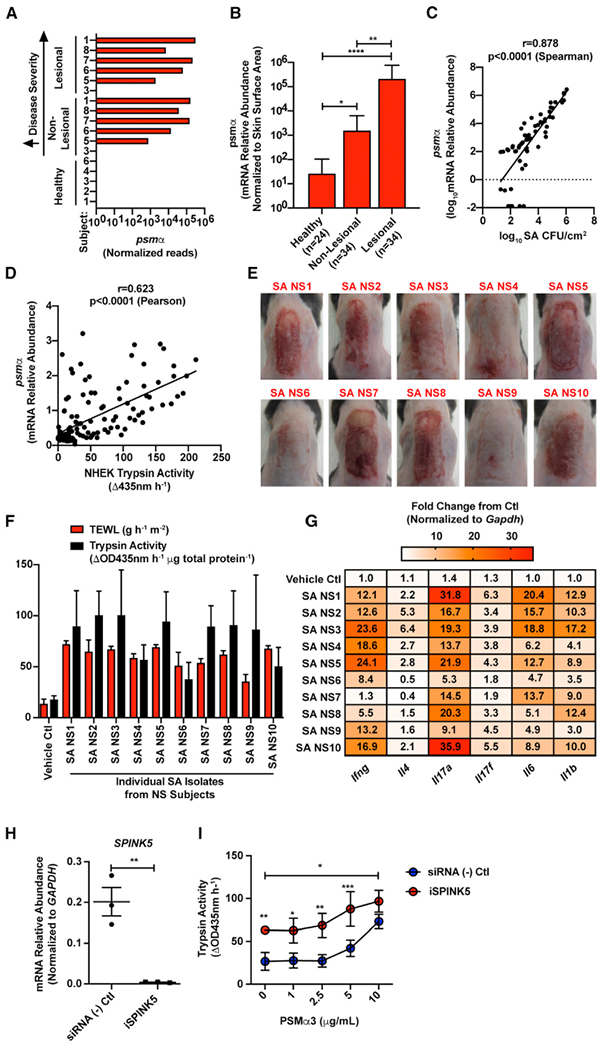
*Staphylococcus aureus* PSMα Is Increased on Netherton syndrome Skin and Promotes Epidermal Protease Activity (A) Normalized counts of the gene *psmα* detected from metagenomic samples. (B) Relative abundance of *S. aureus psmα* mRNA isolated from skin swabs of healthy and NS non-lesional and lesional skin (n, number of swabs assessed per condition). Results represent mean ± SEM, and a non-parametric unpaired Kruskal-Wallis test was used to determine statistical significance: *p < 0.05, **p < 0.01, ***p < 0.001, and ****p < 0.0001. (C) Spearman correlation between *S. aureus* (SA) CFU/cm^2^ and the relative abundance of *S. aureus psmα* mRNA isolated from skin swabs. Each dot represents an individual swab. (D) Pearson correlation between the trypsin activity induced in neonatal human epidermal keratinocytes (NHEKs) after culture for 24 h with 5% supernatant of clinical *S. aureus* (SA) isolates from NS skin and the relative abundance of psmα mRNA level in the same SA isolates. Each dot represents an individual SA isolate. (E–G) Epicutaneous application of 1e^7^ CFU/cm^2^ of *S. aureus* clinical isolates on murine back skin for 48 h (n = 3 per group). For each NS subject, one lesional *S. aureus* isolate with a high *psmα* expression was selected for mouse skin application. (E) Visual representation of murine back skin after 48 h colonization with 1e^7^ CFU/cm^2^ SA isolates. (F and G) Analysis of (F) epidermal barrier damage (TEWL), trypsin activity, and (G) qPCR analysis of inflammatory cytokines stimulated in murine skin by clinical SA NS isolates 1–10. qPCR cytokine levels (*Ifng*, *Il4*, *Il17a*, *Il17f*, *Il6*, and *Il1b*) are normalized to the housekeeping gene *Gapdh*. (H) Relative abundance of *SPINK5* mRNA in NHEKs that were treated with scrambled control or *SPINK5* siRNA (i*SPINK5*) (n = 3 per condition). Each dot represents an individual sample. Results represent mean ± SEM, and Student’s t test was used to determine statistical significance: *p < 0.05, **p < 0.01, ***p < 0.001, and ****p < 0.0001. (I) Trypsin activity from conditioned medium of NHEKs that were pretreated with scrambled control or *SPINK5* siRNA (*iSPINK5*) and then cultured for 24 h with *S. aureus* synthetic PSMα3 peptide (0, 1, 2.5, 5, and 10 μg/mL) (n = 4 per condition). Results represent mean ± SEM, and two-way ANOVA was used to determine statistical significance: *p < 0.05, **p < 0.01, ***p < 0.001, and ****p < 0.0001. In (E)–(I), experiments are representative of two independent experiments. See also Figure S5.

To identify the mechanism by which *S. aureus* could promote disease in NS, we examined the *S. aureus* virulence factor PSMα. This peptide has been shown to induce activity of several serine proteases of the kallikrein (KLK) family in human keratinocytes (Williams et al., 2017,^2019^). We hypothesized that induced expression of human proteases by PSMα might be amplified by the lack of the serine protease inhibitor LEKTI-1 in NS subjects. Our metagenomic analysis showed that DNA for the *psmα* operon was elevated in all samples in which *S. aureus* colonization was present (subjects 1, 6, 7, and 8), and a strong correlation between the presence of *psmα* and disease severity was observed (Figure 3A). *psmα* mRNA extracted from skin swabs also correlated with the abundance of *S. aureus* colonization on NS skin (Figures 3B and 3C). Analysis of individual *S. aureus* colonies further revealed that expression of the *psmα* operon varied between isolates and that this expression correlated with the capacity of an isolate to induce endogenous serine protease activity in primary human keratinocytes (Figure 3D).

To further establish if *S. aureus* isolates from NS skin could damage the skin and therefore participate in disease, we selected one lesional *S. aureus* isolate per subject to apply epicutaneously to murine back skin. As expected, similar to what was observed with *S. aureus* laboratory strains or isolates from AD skin (Byrd et al., 2018; Liu et al., 2017; Nakagawa et al., 2017; Nakatsuji et al., 2016; Williams et al., 2017, 2019), all the tested *S. aureus* isolates induced visual skin inflammation and barrier damage as assessed by increased transepidermal water loss (TEWL) and endogenous trypsin activity (Figures 3E and 3F). Furthermore, skin exposed to *S. aureus* from NS also demonstrated increased mRNA for cytokines linked to general inflammation, including *Il1b, Il6, Il17a/f,* and *Ifng* (Figure 3G). These findings demonstrate that *S. aureus* NS isolates have the capacity to promote skin barrier damage and inflammation *in vivo*. Next, to determine the relevance of the increased expression of *S. aureus psmα* in NS subjects, normal human keratinocytes were exposed to PSMα3 peptide in culture following inhibition of *SPINK5* by small interfering RNA (siRNA). As expected, the NS keratinocyte model lacked *SPINK5* mRNA expression, and this correlated with increased baseline trypsin activity from the cells. However, PSMα3 significantly increased keratinocyte trypsin activity in *SPINK5*-deficient cells (Figures 3H and 3I) above normal cells. These data demonstrate how PSMα peptides from *S. aureus* can exacerbate proteolytic activity in NS skin because of the unopposed induction of epidermal serine protease activity by this bacterial toxin.

### *S. aureus* Cysteine Proteases Staphopain A and B Are Associated with Disease in NS

**Figure 4.**
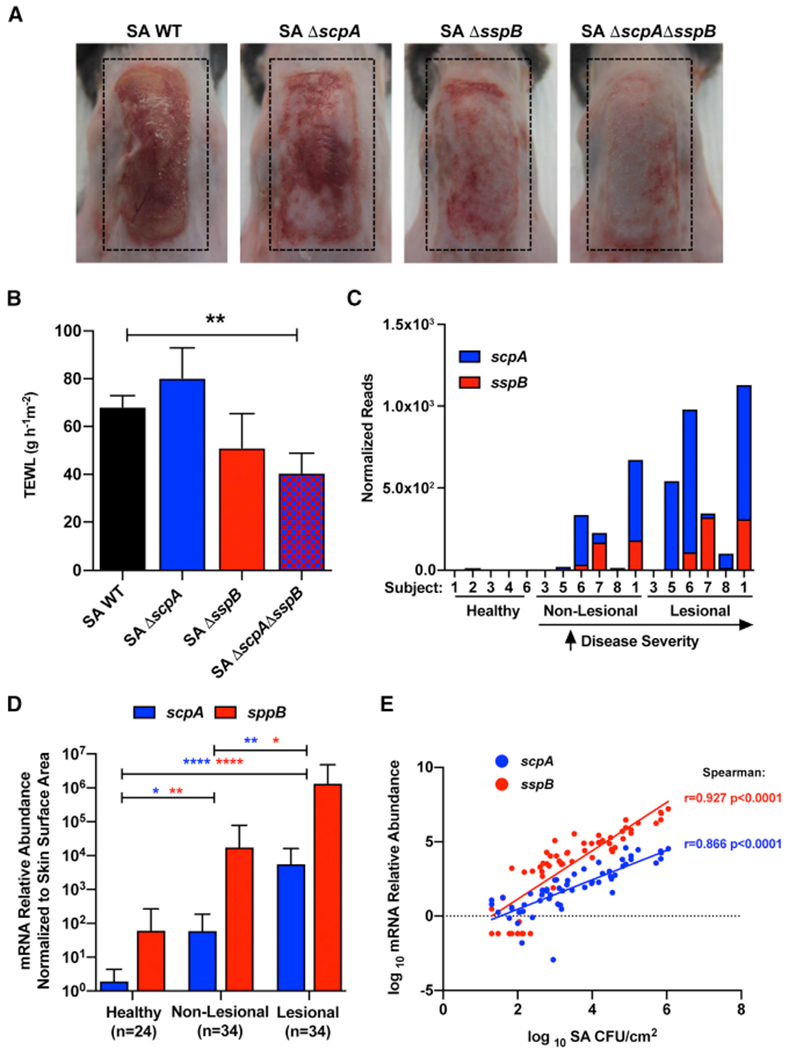
*Staphylococcus aureus* Staphopain A (*scpA*) and B (*sspB*) Are Increased in Netherton Syndrome and Induce Epithelial Barrier Damage (A and B) (A) Representative picture and (B) TEWL measurement of female C57BL/6J murine back skin after epicutaneous application of 1e^7^ CFU/cm^2^ of *S. aureus* (SA) wild-type (WT), SA *scpA* knockout (−Δ*scpA*), SA *sspB* knockout (Δ*sspB*), or SA *scpA*/*sspB* double knockout (Δ*scpA*Δ*sspB*) for 48 h (n = 5 per group). Results represent mean ± SEM, and one-way ANOVA was used to determine statistical significance: *p < 0.05, **p < 0.01, ***p < 0.001, and ****p < 0.0001. (C) Number of reads from metagenomic data corresponding to *S. aureus scpA* (red) and *sspB* (blue) genes normalized per library size for each sample. (D) Relative abundance of *S. aureus scpA* (red) and *sspB* (blue) mRNA isolated from swabs of healthy control and NS non-lesional and lesional skin normalized to skin area (n, number of individual skin swabs per condition). Results represent mean ± SEM, and a non-parametric unpaired Kruskal-Wallis test was used to determine statistical significance: *p < 0.05, **p < 0.01, ***p < 0.001, and ****p < 0.0001. (E) Spearman correlation between the relative abundance of either *scpA* (red) or *sspB* (blue) mRNA and *S. aureus* (SA) CFU/cm^2^ from all skin swabs. Each dot represents an individual swab. Results are represented as mean ± SEM. In (A) and (B), data are representatives of at least two independent experiments. See also Figure S5.

In addition to the keratinocyte protease activity induced by PSMα, staphylococci also secrete several proteases that could interact with LEKTI and participate in damaging the epidermis in NS (Nakatsuji et al., 2016; Williams et al., 2019). To determine the effect of an *S. aureus* protease on skin barrier damage and inflammation, we assessed individual protease gene deletions in a parental strain of *S. aureus* USA300 LAC. Individual deletions of the genes encoding the cysteine proteases staphopain A and B (*scpA*, *sspB*) did not significantly decrease skin damage induced by *S. aureus* after topical application on mouse skin, but a combined knockout of both cysteine proteases reduced inflammation and increased TEWL associated with loss of skin barrier integrity (Figures 4A and 4B). Similar amounts of bacteria were found on mouse skin after treatment, showing that this effect was not due to a difference in levels of colonization by the mutant strains of bacteria (Figure S5). The abundance of gDNA for these cysteine proteases was increased in NS subjects with elevated *S. aureus* abundance in the metagenomic dataset (Figure 4C). In parallel with these findings, both *scpA* and *scpB* showed significantly increased abundance of mRNA on lesional NS skin compared with non-lesional and healthy controls and correlated with the absolute abundance of *S. aureus* found on NS subjects (Figures 4D and 4E). These findings show that *S. aureus* cysteine proteases staphopain A and B can also contribute to skin barrier damage on subjects with NS.

### *S. epidermidis* Cysteine Protease EcpA Is Associated with Disease in NS and Can Promote Skin Damage

**Figure 5.**
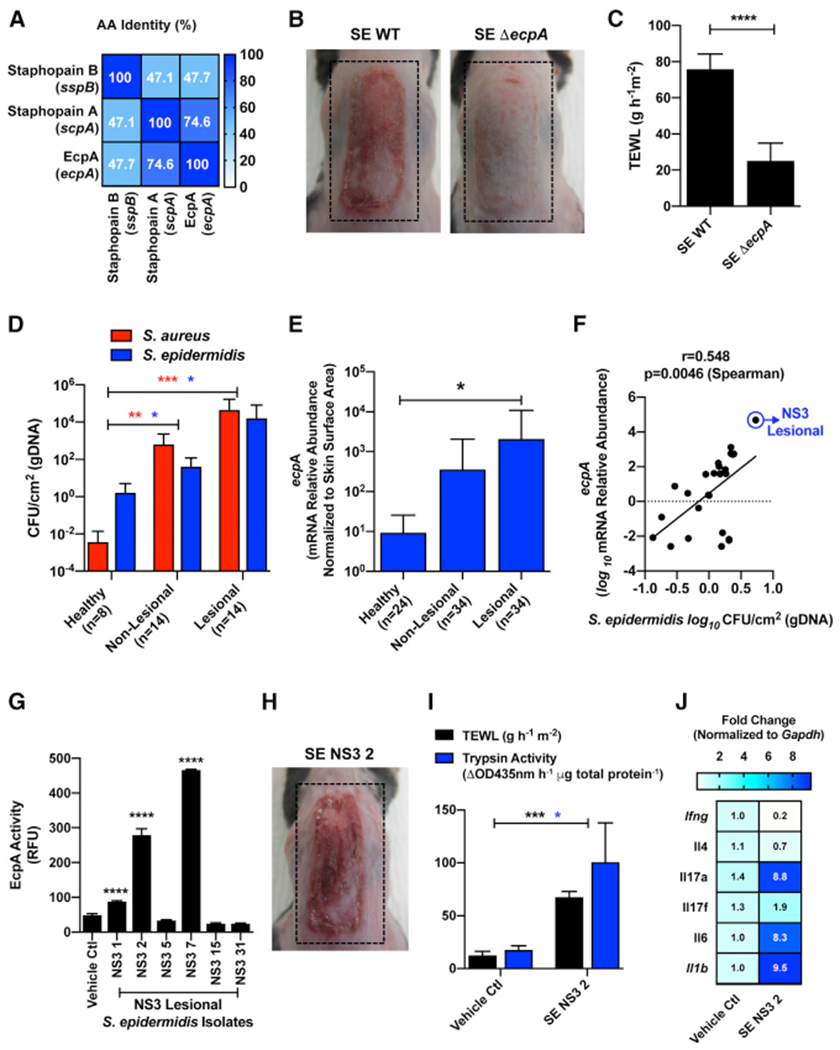
*Staphylococcus epidermidis* Colonization Is Increased in Netherton Syndrome Skin and Can Induce Epithelial Barrier Damage through the Expression of the Cysteine Protease EcpA (A) Percentage amino acid sequence identity of the mature forms of the two *S. aureus* secreted cysteine proteases staphopain A (*scpA*) and staphopain B (*sspB*) and the *S. epidermidis* secreted cysteine protease EcpA (*ecpA*). (B and C) (B) Representative pictures and (C) TEWL measurement of female C57BL/6J murine back skin after epicutaneous application of 1e^7^ CFU/cm^2^ of *S. epidermidis* (SE) wild-type (WT) or SE *ecpA* knockout (Δ*ecpA*) strains for 48h (n = 5 per group). Results represent mean ± SEM, and Student’s t test was used to determine statistical significance: *p < 0.05, **p < 0.01, ***p < 0.001, and ****p < 0.0001. (D) Measurement of gDNA absolute abundance of *S. epidermidis* (blue bars) and *S. aureus* (red bars) CFU/cm^2^ on NS (non-lesional and lesional) versus healthy skin (n, number of individual skin swabs per condition). Results represent mean ± SEM, and a non-parametric unpaired Kruskal-Wallis test was used to determine statistical significance: *p < 0.05, **p < 0.01, ***p < 0.001, and ****p < 0.0001. (E) Relative abundance of *S. epidermidis ecpA* mRNA isolated from swabs of healthy control and NS non-lesional and lesional skin normalized to skin area (n, number of individual skin swabs per condition). Results represent mean ± SEM, and a non-parametric unpaired Kruskal-Wallis test was used to determine statistical significance: *p < 0.05, **p < 0.01, ***p < 0.001, and ****p < 0.0001. (F) Spearman correlation between the relative abundance of *S. epidermidis ecpA* mRNA and *S. epidermidis* CFU/cm^2^ (gDNA) from skin swabs. (G) Assessment of subject NS3 isolated *S. epidermidis* isolates from lesional skin swabs for specific cleavage of EcpA substrate (n = 3). Results represent mean ± SEM, and a one-way ANOVA was used to determine statistical significance: *p < 0.05, **p < 0.01, ***p < 0.001, and ****p < 0.0001. (H) Representative picture of murine back skin after 48 h colonization with 1e^7^ CFU/cm^2^ of clinical *S. epidermidis* isolate NS3 2 (SE. NS3 2). (I and J) Analysis of epidermal barrier damage (TEWL), trypsin activity, and qPCR analysis of inflammatory cytokines stimulated in murine skin by *S. epidermidis* isolate SE. NS3 2. qPCR cytokine levels (*Ifng*, *Il4*, *Il17a*, *Il17f*, *Il6*, and *Il1b*) are normalized to the housekeeping gene *Gapdh*. In (B), (C), and (G)–(J), data are representatives of at least two independent experiments. See also Figures S5 and S6.

Because a predominance of colonization by *S. epidermidis* was also seen in NS, we sought to determine how this species might contribute to disease. A potential role for *S. epidermidis* was particularly evident in NS subject 3 (NS3), who displayed low levels of *S. aureus* colonization but elevated *S. epidermidis* relative to controls (Figures 2A and 2C). *S. epidermidis* is known to secrete a single cysteine protease termed extracellular cysteine protease A (EcpA) that has similarity to *S. aureus* cysteine proteases staphopain A (ScpA) and staphopain B (SspB) (Dubin et al., 2001; Oleksy et al., 2004). Alignment of amino acid sequences of the active cysteine proteases showed homology between *S. aureus* staphopains A and B and *S. epidermidis* EcpA (Figure 5A; Figure S6). Using a single-gene deletion knockout for the EcpA gene (*ecpA*) in *S. epidermidis*, we established that *S. epidermidis* can also drive skin barrier damage and inflammation on murine back skin through the expression of this cysteine protease (Figures 5B and 5C). The difference of effect between the two bacteria was not due to a different level of colonization (Figure S5A). Analysis of the human skin swab samples showed increased *S. epidermidis* gDNA absolute abundance levels on both non-lesional and lesional skin that were comparable to increases in *S. aureus* compared with healthy control subjects (Figure 5D). Furthermore, mRNA abundance for *ecpA* found on NS skin swabs was significantly elevated on lesional skin, and the relative abundance correlated with the gDNA absolute abundance of *S. epidermidis* found on the skin (Figures 5E and 5F). These data revealed that subject NS3 had the highest abundance of both *S. epidermidis* and *ecpA* mRNA and displayed the least amount of live *S. aureus* (Figure 2C). The analysis of individual *S. epidermidis* isolates from subject NS3 then revealed that activity of EcpA varied between isolates (Figure 5G). To determinate if *S. epidermidis* isolates from NS skin could damage the skin, an *S. epidermidis* isolate selected for having EcpA activity was applied on mice. Similar to what was observed with *S. aureus* clinical isolates, this *S. epidermidis* isolate induced skin damage measured by increased TEWL and increased endogenous trypsin activity (Figures 5H and 5I). Moreover, *S. epidermidis* isolate NS3 2 also increased the mRNA levels of *Il1b*, *Il6*, and *Il17a/f* but not *Ifng* or *Il4* (Figure 5J). These findings suggest that *S. epidermidis* can contribute to skin damage when present on NS skin through the expression of the cysteine protease EcpA.

## DISCUSSION

A fundamental understanding of how commensal microbial gene products interact with the human genome is generally lacking. In this study, we focused on subjects with an autosomal-recessive skin disease that results from loss-of-function mutations in a single gene, the serine protease inhibitor *SPINK5.* This syndrome was of interest because although it is a germline mutation, the severity and location of inflammation vary over time, thus suggesting the potential that this single-gene mutation enables a dynamic epidermal microbial community to influence the observed phenotype. Bacteria present on subjects with NS were similar to one another but differed greatly from bacteria present on the skin of healthy control subjects. Staphylococcal species, in particular either *S. aureus* or *S. epidermidis*, were predominant on NS subjects. Genomic and functional analysis identified specific gene products from these bacteria that altered the proteolytic activity of the epidermis and therefore were relevant to subjects with loss of *SPINK5.* This human disease demonstrates how products of the human and microbial genomes can interact and illustrates the functional consequences of loss of homeostasis driven by a defect in this system. The epidermal ecosystem is multidimensional, and our findings show how several bacterial genes from different species can contribute to a disease phenotype. Overall we provide an important model of how communication with commensal microbes influences human health.

This study presents one of the most deeply sequenced skin microbiome datasets to date, which has allowed metagenomic co-assembly. The QUAST analysis of the co-assemblies shows that the major shared genomes were highly covered, as does the *psmα* contig analysis. The strain resolved phylogenetic profiling was conducted using software explicitly designed for this analysis (Nayfach et al., 2016). Despite the findings of the metagenomic sequencing presented in this study, we do recognize several potential shortcomings. As with other skin microbiome studies, a lack of ability to remove the majority of human DNA contamination leads to difficulty in assessing bacteria reads with enough coverage for assembly. Sampling of the NS subject cohort was also not contained to a single common site, because of the sporadic nature of NS skin lesions. Skin swabs were collected based upon the availability of lesions at each visit. These swab sites were predominately on the abdomen and local extremities (e.g., shoulder, arm, thigh), which all have similar baseline healthy microbiomes and are thus relevant to compare. Age can also lead to varying skin microbiome populations. It has been observed that younger patients have significantly less *Propionibacterium* on the skin surface than older patients (Oh et al., 2012). Subject ages in this study varied drastically, ranging from 12 to 47 years, with the presence of younger subjects making it difficult to include age-matched healthy control subjects. Despite these challenges in sample collection with a rare skin disease, all sites sampled displayed a clear phenotype of increased staphylococci colonization that differed greatly from normal skin (age range 27–52 years) and often was predominated by *S. aureus.* This does not occur in the healthy population despite age or swab site, and the percentage of NS individuals who were positive for *S. aureus* (>90%) greatly exceeded how often AD subjects are colonized by *S. aureus* (>50%). Overall, these findings provide a highly informative deep shotgun sequencing metagenomic analysis of a skin disease and suggest that the gene mutation in NS results in a unique skin microbiome.

Several of the observations made in this study were unexpected. *S. aureus* was known to promote damage to keratinocytes and induce skin inflammation (Liu et al., 2017; Nakagawa et al., 2017; Nakatsuji et al., 2016; Williams et al., 2017, 2019) but was not known to play such an important role in NS. Although the presence of *S. aureus* on NS subject skin was already reported in the literature (Chao et al., 2005; Renner et al., 2009; Śmigiel et al., 2017), the present study showed that NS subjects appeared to be almost universally colonized by *S. aureus*. Furthermore, we demonstrated that *S. aureus* isolates from NS subjects have similar effect on mouse skin to what was previously observed with isolates from AD skin (Williams et al., 2019). PSMα from isolates of both diseases induced serine protease activity and promoted skin damage. Moreover, *S. aureus* staphopain A (*scpA*) and staphopain B (*sspB*), two cysteine proteases previously underappreciated for their virulence capacity, were shown to contribute to this response. Human keratinocytes showed increased protease activity when *SPINK5* was silenced and treated with PSMα3, thus showing in human cells how the loss-of-function mutations in *SPINK5* found in NS subjects will increase their susceptibility to proteolytic damage promoted by *S. aureus*. In agreement with our data, a case report showed a very significant improvement of symptoms and quality of life of a subject with NS after treatment with an anti-staphylococcal bacteriophage preparation (Zhvania et al., 2017). Altogether, these findings demonstrate how *S. aureus* present on NS skin is a major factor that exacerbates the pathogenesis of NS.

An important and unexpected observation from this study was also that *S. aureus* is not the only member of the complex skin microbiome that promotes inflammation in NS. Metagenomic analysis of NS subjects found an increase in the relative abundance of *S. epidermidis*, with one subject being preferentially dominated by *S. epidermidis*. We demonstrated that an *S. epidermidis* NS isolate was able to induce skin damage in mice. This finding was very surprising, as *S. epidermidis* was previously regarded as one of the key members of the normal skin microbiota and not recognized to have pathogenic effects when colonizing only the skin surface. In contrast, *S. epidermidis* has been hypothesized to have multiple beneficial effects for the host (Byrd et al., 2018; Nakatsuji and Gallo, 2019; Stacy and Belkaid, 2019). Various *S. epidermidis* strains have been shown to have the capability to tune skin immunity, to promote wound repair, to limit pathogen infections, and to protect against skin tumors (Lai et al., 2009, 2010; Linehan et al., 2018; Naik et al., 2015; Nakatsuji et al., 2017, 2018). Under some conditions, such as those that were apparently present on NS3, *S. epidermidis* may effectively outcompete *S. aureus* for dominance on the skin (Nakatsuji et al., 2017). Although *S. epidermidis* can also be an invasive pathogen (Dong et al., 2018; Le et al., 2018; Otto, 2009; Uçkay et al., 2009), this behavior is opportunistic and occurs typically in a setting of immunosuppression or implanted foreign devices.

The cysteine protease EcpA was shown to be a major factor used by *S. epidermidis* to damage the skin, as its deletion in a laboratory strain prevented this effect, and several clinical isolates expressing EcpA had a similar capacity to promote epidermal injury. It is unlikely that the capacity of *S. epidermidis* to induce skin inflammation is specific to NS isolates. As EcpA expression is under the control of the accessory gene regulatory *(agr)* quorum sensing system of *S. epidermidis* (Olson et al., 2014), we speculate that either the density of *S. epidermidis* on normal skin is too low to activate the agr quorum sensing system, or other commensal coagulase-negative staphylococci can inhibit its agr system when the relative abundance of *S. epidermidis* is low. This could explain why on healthy skin *S. epidermidis* does not produce sufficient EcpA to induce disease. Alternatively, LEKTI-1 has also been shown to have the capacity to inhibit cysteine proteases (Bennett et al., 2010). Thus, in NS subjects, the loss of a single gene expressing LEKTI-1 could lead to a skin microenvironment that promotes excess activity of EcpA. This may occur in association with *S. aureus*, or in some cases, such as subject NS3, skin lesions may be induced by *S. epidermidis* acting alone. The contribution of other bacterial proteases or toxins to this system cannot be excluded and are considered to be likely (Cheung et al., 2014; Qin et al., 2017; Queck et al., 2009).

NS has similarities to AD but also has important differences. For instance, NS subjects show large dysbiosis across both non-lesional and lesional skin, while AD subjects mostly display dysbiosis only on lesional sites (Byrd et al., 2017). AD subjects display increased *S. epidermidis* and *S. aureus* skin colonization during disease flares (Byrd et al., 2017), a response similar to what we observed here on NS subjects. Interestingly, all NS subjects in this study revealed high amounts of *S. aureus*, but many AD subjects are not culture positive for *S. aureus*. Interestingly, our findings show how “commensal” *S. epidermidis* strains may behave on the skin in a similar way to *S. aureus* to disrupt proteolytic balance and promote inflammation. Therefore, consideration should also be given to *S. epidermidis* as a pathogen in AD. Further research into the negative effects of *S. epidermidis* in AD and other disorders is needed.

This study of subjects with NS provides insight into host genetic mechanisms behind changes in the human skin microbiome. In the case of NS, loss of a single serine protease inhibitor is enough to enable dysbiosis, but it is unclear why normal appearing skin on NS subjects also has an abnormal microbiome. Other host factors may be involved in control of the microbial community on the skin. The presence of the gene products identified here from *S. aureus* and *S. epidermidis* appears to contribute to the pathogenesis of skin lesions in the adult subjects, but it does not answer why dysbiosis is established. We hypothesize that the proteolytic imbalance from loss of *SPINK5* influences innate immune function and the physical barrier. For example, serine proteases promote activation and inactivation of the antimicrobial peptide LL-37 (Yamasaki et al., 2006). LL-37 is an important antimicrobial peptide in the skin against *S. aureus* (Larrick et al., 1995; Noore et al., 2013). Elevated serine protease activity promotes cleavage of LL-37 (Morizane et al., 2010). Once *S. aureus* colonization is established, its own secreted proteases can also degrade LL-37 (Sieprawska-Lupa et al., 2004; Sonesson et al., 2017). Thus, it is possible that elevated endogenous serine protease activity due to loss of *SPINK5* can prevent host immunity from establishing a healthy homeostasis. As the epidermis matures, established changes to the microbial ecosystem can then further enable manifestations of disease in NS in a localized and transient pattern. Because LEKTI-1 deficiency is lethal in mice (Descargues et al., 2005), efforts are currently being made to develop a viable mouse model for NS to further investigate this question.

In conclusion, this work illustrates how the rare monogenetic skin disease NS results in the establishment of a distinct bacterial community on the skin of adult subjects that is dominated by *S. aureus* and *S. epidermidis*. Our data suggest that specific gene products from these microbes may promote the barrier disruption that is characteristic of this disorder. These observations reveal an unanticipated pathway for *Staphylococci* to contribute to skin inflammation in this disease. Importantly, this work also illustrates how a single genetic modification in a host extracellular protease inhibitor affects the complex ecosystem of the skin. These observations uncover potential areas for future therapeutic interventions in NS.

## STAR★METHODS

### LEAD CONTACT AND MATERIALS AVAILABILITY

Further information and requests for resources and reagents should be directed to and will be fulfilled by the lead contact, Richard Gallo (rgallo@ucsd.edu). All bacteria strains used in this study are available from the lead contact while the metagenomic dataset is deposited to NCBI and available according the Bioproject number PRJNA551026.

### EXPERIMENTAL MODELS AND SUBJECT DETAILS

#### Human subjects

All experiments involving human subjects were sponsored by INSERM Institute (C12-56, ID-RCB Number: 2013-A00275-40) and were carried out according to protocols approved by the French Agence Nationale de Sécurité du Médicament (ANSM; Project #131066B42) and by the Institutional Review Board (Project #101-13). Skin swabs from 10 subjects with NS (non-lesional and lesional; ages ranging from 12-47 years old listed in Table S1) and 8 healthy subjects (ages ranging from 27-52 years old listed in Table S2) were used in this study (Table S1). Both NS and healthy groups included both male and female subjects. NS subjects were confirmed clinically according to at least 3 of the following parameters: (i) scaly erythroderma in the first year of life, (ii) ichthyosis lineariz circumflexa; (iii) hair shaft defect including trichorrhexis invaginata, and (iii) atopic manifestations (elevated total IgE serum levels). Second, NS subjects were confirmed due to analysis of subject mutations to the *SPINK5* gene by direct sequencing of the coding region and adjacent intronic sequences of *SPINK5* as reported previously (Bitoun et al., 2002). To compare the severity between individuals, a severity score (Figure S1) based on the following criteria: scaly erythroderma and ichthyosis lineariz circumflexa (absent = 0, minimum = 1, moderate = 2, severe = 3, very severe = 4) was used.

#### Bacteria preparation

All *S. aureus* and *S. epidermidis* strains were grown overnight (18h) to stationary phase in 3% tryptic soy broth (TSB) at 300RPM in a 37°C incubator unless stated otherwise. This growth time for all staphylococci indicated approximately an OD600nm reading of 10 and 1e^9^ CFU. For treatment of bacterial supernatant on human keratinocytes, overnight cultured bacteria were pelleted (15min, 4,000RPM, RT) followed by filter sterilization of the supernatant (0.22 μm). For mouse experiments with live bacteria colonization, bacterial CFU was approximated by OD600nm prior to application to mouse back skin followed by confirmation of the actual CFU the following day.

#### Mouse model of epicutaneous bacteria exposure

Age-matched 8-10 weeks old female C57BL/6J mice were used in all experiments (n = 3-5 per condition). Mice were co-housed with 3-5 mice per cage in all experiments. All animal experiments were approved by the UCSD (University of California, San Diego) Institutional Animal Care and Use Committee (Protocol#S09074). A previously described mouse model of epicutaneous bacterial exposure was used (Williams et al., 2019). Briefly, the dorsal skin of anesthetized mice (2% isoflurane) was shaved and depilated using Nair cream for 2min followed by immediate removal with sterile alcohol wipes. The skin barrier was allowed to recover from hair removal for 24h prior to application of bacteria. *S. aureus* and *S. epidermidis* (1e^7^ CFU) in 3%TSB was applied to murine skin for 48h at a 100 μL volume on a 2x1cm piece of sterile gauze. A bio-occlusive dressing (Tegaderm; 3M) along with a flexible fabric Band-Aid was applied on top of gauze to hold in place for duration of the treatment.

#### Normal human keratinocyte model

Normal neonatal human epidermal keratinocytes (NHEKs; Thermo Fisher Scientific) were cultured in Epilife complete medium containing 60 μM CaCl_2_ (Thermo Fisher Scientific) supplemented with 1x Epilife Defined Growth Supplement (EDGS; Thermo Fisher Scientific) and 1x antibiotic-antimycotic (PSA; 100U/mL penicillin, 100U/mL streptomycin, 250ng/mL amphotericin B; Thermo Fisher Scientific) at 37°C, 5%CO2. NHEKs were only used for experiments between passages 3-5. NHEKs were grown to approximately 80% confluency followed by differentiation in high calcium (2mM CaCl_2_) EpiLife complete medium for 48h. For bacterial supernatant treatments, differentiated NHEKs were treated with sterile-filtered bacterial supernatant at 5% by volume to Epilife medium for 24h.

#### SPINK5 gene silencing human keratinocyte model

NHEKs grown to 50% confluency were treated for 24h with 20nM *SPINK5* silencer select siRNA or a siRNA scrambled (-) control (ThermoFisher Scientific) using Lipofectamine RNAiMAX Transfection Reagent (Invitrogen) and OptiMEM medium (GIBCO). After 24h, remaining siRNA was removed and NHEKs were supplemented with fresh Epilife complete medium for 48h until cells reached about a complete monolayer followed by addition high calcium Epilife complete medium 2mM CaCl_2_ for an additional 48h. Finally, *S. aureus* synthetic PSMα3 peptide (10 μg/mL) was added to differentiated NHEKs for an additional 24h prior to analysis.

### METHOD DETAILS

#### Collection of skin microbiome from human subjects

For all subjects with Netherton syndrome, collection of surface bacteria was done from a pre-measured area (15cm^2^) of both lesional and non-lesional skin, on different anatomical areas according to lesion location and at different time points (Table S1). For the healthy subjects, surface bacteria were similarly collected on matching anatomical areas (Table S2). For collection of skin microbiome DNA, swab head was soaked in 1mL of molecular biology grade TE buffer (Invitrogen) containing 0.1% Triton X-100 and 0.05% Tween-20 and surface bacteria were collected by rubbing the pre-measured skin area with 50 strokes of constant pressure. Swab head was placed in a microcentrifuge tube and immediately stored at −80°C for further analysis. For collection of skin microbiome RNA and live bacteria, swab head was soaked in 3%TSB and 16.67% glycerol solution and surface bacteria were collected as described above. Swab head was placed in a microcentrifuge tube containing 1 mL of 3%TSB and 16.67% glycerol solution. Following 1 min of vortexing samples, the bacterial suspensions were stored at −80°C for further analysis. After skin microbiome collection, the skin of subjects was cleaned with an alcohol swab.

### Microbiome metagenomic analysis

#### DNA extraction, library preparation, and shotgun sequencing

Microbiome DNA was extracted from skin swab using the PureLink Microbiome DNA Purification Kit according to manufacturer’s instructions (Thermo Fisher Scientific). After purification, samples were treated with NEBNext microbiome DNA enrichment kit according to manufacturer’s instructions (New England Biolabs) except that SPRI select beads (Beckman Coulter) were used for the final clean-up step and 40μL of Low EDTA TE buffer (Swift Biosciences) to elute the samples. The concentration of samples was monitored before and after enrichment using Qubit dsDNA HS assay kit (Invitrogen). 300bp fragments were generated by sonication. Next-generation sequencing (NGS) libraries were then prepared using Accel-NGS 2S Plus DNA Library Kit and 2S Indexed Adaptor Kit according to manufacturer’s instructions (Swift Biosciences) and using SPRI select beads (Beckman Coulter) for all clean-up steps. Before indexing, the quality and fragment-size of the libraries were checked using High Sensitivity D1000 ScreenTape according to manufacturer’s instructions (Agilent Technologies). Finally, HiSeq 2500 150bp paired-end sequencing (Illumina) was performed. 17 samples in total (12 NS lesional (n = 6) and non-lesional (n = 6) and 5 healthy controls) were selected for metagenomic sequencing based upon < 98% human DNA contamination. Cumulatively, the total number of reads across all samples was 2.6x10^8^ reads (with an average of 1.5x10^7^ reads per sample.

#### Quality control, host removal and metagenomic co-assembly

Raw metagenomic reads were trimmed for quality by removing low quality reads, followed by removal of reads that mapped to the human genome (assembly19) using KneadData (version 0.5.4) with default parameters. Post quality trimming, the percentage of host contamination among subjects ranged from < 10% in some samples to > 80% in a few others, accounting for an average of approximately 1x10^7^ reads per sample. The total number of reads after discarding those that mapped back to the human reference was 4.1x10^7^, with an average of 2.4x10^6^ reads per sample. The host decontaminated reads from all samples (skin conditions including both NS syndrome (non-lesional and lesional) and healthy samples) were then co-assembled using metaSPAdes (version 3.11.1) on k-mer sizes of k = 21,33,55,77. Furthermore, reads across all samples were also combined and co-assembled on the basis of skin condition; i.e., healthy, lesional, and non-lesional to compare total size for the assembled cohorts and assess cumulative genome length and subsequent genome overlap between them (Table S3).

#### Taxonomic composition using species and strain level profiling

Species level taxonomic profiling was performed on the post-processed reads in order to examine the community composition using MIDAS (version 1.3.0) by leveraging approximately 30,000 bacterial reference genomes, clustered into 5,952 species groups. Relative taxonomic abundances were estimated for each of the bacterial species groups, or strains, across 30 universal single copy marker genes included in the marker_genes reference database by mapping reads to gene-specific, species-level mapping thresholds (94.5%–98% nucleotide identity). Hierarchical clustering was performed for the top 40 most prevalent species were visualized for the entire cohort using “dplyr” to filter the data frame and “heatmap3” with the “average” linkage method for generating the heatmap in R (version 3.4.1). MIDAS also allowed for pan-genomic profiling by mapping metagenomic reads to species of interest to quantify the abundance of these pangenomic genes, with at least one mapped read, across all samples. Results were visualized using “dplyr” and “reshape2” to manipulate the data and “ggplot2” for generating the plots in R.

#### Community richness and diversity based on ordination analyses

Microbial community richness and diversity metrics were examined using multidimensional scaling techniques like Principal Coordinates Analysis (PCoA) on the read counts matrix previously generated by taxonomic species level profiling for all samples. Beta diversity metrics like bray-curtis dissimilarity index and alpha diversity metrics like shannon, chao1 and observed_otus were calculated on reads from all species that had an abundance of at least 1.0 across all samples. These metrics were then explored and visualized in QIIME 2 (version 2019.1). Between and within beta diversity based on sample groupings by skin condition (healthy/non-lesional/lesional) and subject code was also explored.

#### Identification of staphylococcal virulence factors among subjects

Each contig from the all-inclusive co-assembly was screened for the presence and expression of specific virulence factors produced by *S. aureus* and *S. epidermidis* that are vital for pathogenicity, specifically the following six genes: (1) Phenol-soluble modulin α (PSMα1: fMGIIAGIIKVIKSLIEQFTGK (Williams et al., 2019) (2) Staphopain A *(scpA* [GenBank: CAD61962.1]) (3) Staphopain B *(sspB* [GenBank: AAG45844.1]) (4) Extracellular cysteine protease (*ecpA* [GenBank: AJ298299]. Assembly ORFs were called on all contigs using FragGeneScan (version 1.16), and read counts for each ORF across all samples were obtained by mapping reads to predicted ORFs using clc_ref_assemble_long in CLC Assembly Cell (CLC bio, version 3.22.55705) using the method as described in (Dupont et al., 2015). These ORFs were also searched against PhyloDB (version 1.076) using Blast (E-value threshold < 1e-03) to establish phylogenetic annotation. Additionally, the ORFs were also searched against the Pfam and TIGRfam custom database using HMMER (version 3.0) to establish functional annotation. For Pfam and TIGRFAM assignments, only matches with scores above the model trusted cut-off score were considered. Finally, all phylogenetic and functional annotation results were merged with the read counts for all ORFs across all samples.

#### Quantification of live staphylococci and collection of isolates from skin swabs

For quantification of live staphylococci and collection of clinical isolates, swabs were rapidly thawed, vortexed, serial diluted and plated onto mannitol salt agar (MSA) selection plates supplemented with 3% egg yolk. After overnight incubation at 37°C, the number of colony-forming unit (CFU) was determined. *S. aureus* was distinguished from coagulase-negative staphylococci (CoNS) according to mannitol metabolism and the egg yolk reaction as described previously (Iwase et al., 2010; Nakatsuji et al., 2017). For each swab, various CoNS and *S. aureus* isolates were picked, cultured overnight at 37°C in 3%TSB and stored at −80°C in 3%TSB and 16.67% glycerol solution for further analyses.

#### Quantification of staphylococcal genomic DNA (gDNA)

The absolute abundance of *S. aureus* and *S. epidermidis* gDNA in the microbial DNA elution prepared from skin swabs was determined by quantitative real-time PCR (qPCR) as previously described (Nakatsuji et al., 2013, 2016). Briefly, qPCR was performed with Power SYBR Green Master mix (Applied Biosystems) using *S. epidermidis and S. aureus* specific primers, targeting the *S. epidermidis gseA* gene and *S. aureus femA* gene respectively. To determine the relative CFU of *S. aureus* or *S. epidermidis* specific DNA, a standard curve was generated with gDNA extracted from known CFUs of *S. aureus* (ATCC113) or *S. epidermidis* (ATCC12228), respectively. The specificity of all primer pairs was confirmed by melting curve analysis and comparison with standard curves.

#### Transepidermal water loss measurement

To determine damage to the epidermal skin barrier, transepidermal water loss (TEWL) of murine skin treated for 48h with *S. aureus* or *S. epidermidis* was measured using a TEWAMETER TM300 (C & K).

#### RNA isolation and quantitative real-time PCR

All RNA was isolated using the Purelink RNA isolation kit according to manufacturer’s instructions (Thermo Fisher Scientific). For mouse tissue, about 0.5cm^2^ full thickness skin was bead beat in 750 μL of RNA lysis buffer (2x 30sec with 5min on ice after each, 2.0mm zirconia bead). Tissue was then centrifuged (10min, 13,000RPM, 4°C), followed by adding 350 μL of clear lysate to 70% EtOH and column based isolation of RNA. For isolation of human skin swab microbiome RNA, skins swabs were vortexed (30sec) followed by incubation of 250 μL with RNAprotect reagent (QIAGEN) for 10min prior to centrifugation (10min, 13,000RPM, RT), resuspension in 750 μL of RNA lysis buffer, and bead beating (2x 1min with 5min on ice after each) using lysing matrix B tubes. Samples were then centrifuged again and 350 μL of clear lysate was added to 70% EtOH as above. After RNA isolation, samples were quantified with a Nanodrop (ThermoFisher Scientific), and 500ng of bacterial or mouse RNA was reverse-transcribed using the iScript cDNA synthesis kit (Bio-Rad). qPCR reactions were ran on a CFX96 Real-Time Detection System (Bio-Rad). For mouse samples, gene-specific primers and TaqMan probes (Thermo Fisher Scientific) were used with GAPDH as a housekeeping gene. For bacterial RNA, SYBR Green qPCR Master Mix (Biotool) was used along with specific primers. The mRNA relative abundance of the genes *sspB*, *scpA* and *ecpA* were normalized to the overall skin area swabbed.

#### NHEK and murine skin trypsin activity analysis

NHEK conditioned medium was added at 50 μL to black 96 well black bottom plates (Corning) followed by addition of 150 μL of the peptide Boc-Val-Pro-Arg-AMC (trypsin-like substrate, BACHEM) at a final concentration of 200 μM in 1x digestion buffer (10 mM Tris-HCl pH7.8, Teknova) and incubated at 37°C for 24h. Relative fluorescent intensity (excitation: 354nm, emission: 435nm) was analyzed with a SpectraMAX Gemini EM fluorometer (Thermo Fisher Scientific). For murine skin trypsin activity analysis, 0.5cm^2^ full-thickness skin was bead beat (2.0mm zirconia beads, 2x 30sec with 5min on ice after each) in 1mL of 1M acetic acid followed by an overnight rotation at 4°C. Samples were centrifuged (10min, 13,000RPM, 4°C), added to a new microcentrifuge tube followed by protein concentration using a speedvac to remove all remaining acetic acid. Proteins were re-suspended in molecular grade water (500 μL) and rotated overnight at 4°C followed by another centrifugation. Clear protein lysates were added to a new tube, and BCA (Bio-rad) analysis used to determine protein concentration. Finally, 10 μg of total protein was added to a 96 well plate followed by analysis with the trypsin substrate as above.

#### Staphylococcus epidermidis protease EcpA activity analysis

For the measurement of *S. epidermidis* protease activity, bacteria were grown for 24h in 3%TSB at 37°C before preparation of filtered-sterilized supernatant. The supernatant was tested for ecpA activity using a specific FRET substrate with the sequence (5-FAM)-Lys-Leu-Leu-Asp-Ala-Ala-Pro-Lys-(QXL520)-OH (AnaSpec, Fremont, CA) (Olson et al., 2014). 25 μl of *S. epidermidis* supernatant was added to black 96 well black bottom plates (Corning) followed by addition of 25 μL of 1x digestion buffer (10 mM Tris-HCl pH7.8, Teknova) containing the ecpA FRET substrate (1nM final). Relative fluorescent intensity (excitation: 485nm, emission: 538nm) was measured with a SpectraMAX Gemini EM fluorometer (Thermo Fisher Scientific) at t = 0 and after incubation at 37°C for 24h.

#### Mature cysteine protease Staphopain A, Staphopain B and EcpA sequence alignment

The amino acid sequence of the mature forms of the two *S. aureus* cysteine proteases Staphopain A (UniProtKB/Swiss-Prot: P81297.2) and Staphopain B (UniProtKB/Swiss-Prot: P0C1S6.1) and the *S. epidermidis* cysteine protease EcpA (UniProtKB/Swiss-Prot: P0C0Q0.1) were aligned using Geneious R11.1.5 (https://www.geneious.com).

### QUANTIFICATION AND STATISTICAL ANALYSIS

All figures utilized non-parametric unpaired Kruskal Wallis analysis, Student’s t tests, and One/Two-way ANOVAs for statistical analysis as indicated in the figure legends. All statistical analysis was performed using GraphPad Prism Version 8.0 (GraphPad, La Jolla, CA). All data is presented as mean ± standard error of the mean (SEM) and a P value ≤ 0.05 considered significant.

### DATA AND CODE AVAILABILITY

The accession number for the Netherton syndrome high-throughput shotgun sequencing metagenomic datasets generated in the course of this project have been deposited at the National Center for Biotechnology Information Sequence Read Archive under BioProject ID: PRJNA551026. Any further details regarding these datasets will be made available upon request.

## Supplementary Material

1

## Figures and Tables

**Table:** KEY RESOURCES TABLE

REAGENT or RESOURCE	SOURCE	IDENTIFIER
Bacterial and Virus Strains		
*Staphylococcus aureus* NS1	Gallo (UCSD)	This Study
*Staphylococcus aureus* NS2	Gallo (UCSD)	This Study
*Staphylococcus aureus* NS3	Gallo (UCSD)	This Study
*Staphylococcus aureus* NS4	Gallo (UCSD)	This Study
*Staphylococcus aureus* NS5	Gallo (UCSD)	This Study
*Staphylococcus aureus* NS6	Gallo (UCSD)	This Study
*Staphylococcus aureus* NS7	Gallo (UCSD)	This Study
*Staphylococcus aureus* NS8	Gallo (UCSD)	This Study
*Staphylococcus aureus* NS9	Gallo (UCSD)	This Study
*Staphylococcus aureus* NS10	Gallo (UCSD)	This Study
*Staphylococcus aureus* USA300 WT (AH1263)	Horswill (UC Denver)	Mootz et al., 2015
*Staphylococcus aureus* USA300 Δ*scpA* (AH1825)	Horswill (UC Denver)	Mootz et al., 2015
*Staphylococcus aureus* USA300 Δ*sspB* (AH2594)	Horswill (UC Denver)	Mootz et al., 2015
*Staphylococcus aureus* USA300 Δ*scpA*Δ*sspB* (AH2595)	Horswill (UC Denver)	Mootz et al., 2015
*Staphylococcus epidermidis* NS3 1	Gallo (UCSD)	This Study
*Staphylococcus epidermidis* NS3 2	Gallo (UCSD)	This Study
*Staphylococcus epidermidis* NS3 5	Gallo (UCSD)	This Study
*Staphylococcus epidermidis* NS3 7	Gallo (UCSD)	This Study
*Staphylococcus epidermidis* NS3 15	Gallo (UCSD)	This Study
*Staphylococcus epidermidis* NS3 31	Gallo (UCSD)	This Study
*Staphylococcus epidermidis* 1457 (AH2490)	Horswill (UC Denver)	Olson et al., 2014
*Staphylococcus epidermidis* 1457 *ΔecpA* (AH2924)	Horswill (UC Denver)	Olson et al., 2014
Chemicals, Peptides, and Recombinant Proteins		
1M Tris-HCl, pH 7.8 solution	Teknova	Cat# T1078
2-mercaptoethanol	Sigma-Aldrich	Cat# M6250
Antibiotic-Antimycotic (100X)	GIBCO	Cat# 15240062
Bacto Agar	BD Biosciences	Cat# 214010
Boc-Val-Pro-Arg-AMC hydrochloride salt	BACHEM	Cat# I-1120
Calcium chloride dihydrate	Sigma-Aldrich	Cat# 22,350-6
Defined Trypsin Inhibitor (DTI)	GIBCO	Cat# R-007-100
DPBS	GIBCO	Cat# 14190-144
EpiLife Defined Growth Supplement (EDGS)	GIBCO	Cat# S-012-5
EpiLife complete medium, with 60 M calcium	GIBCO	Cat# MEPI500CA
Ethyl alcohol, Pure	Sigma-Aldrich	Cat# E7023
Formalin	Azer Scientific	Cat# PFNBF-20
Lipofectamine RNAiMAX Transfection Reagent	Invitrogen	Cat# 13778030
Low EDTA TE buffer	Swift Biosciences	Cat# 90296
Lysing Matrix B	MP Biomedical	Cat# 116911050-CF
Molecular biology grade TE buffer	Invitrogen	Cat# AM9849
OptiMEM medium	GIBCO	Cat# 31985062
PSMα3peptide:fMEFVAKLFKFFKDLLGKFLGNN	LifeTein	N/A
RNAlater Stabilization Solution	Invitrogen	Cat# AM7021
RNAprotect Bacteria Reagent	QIAGEN	Cat# 76506
SPRI Select beads	Beckman Coulter	Cat# B23317
Tryptic soy Broth (TSB)	Sigma-Aldrich	Cat# T8907-1KG
Trypsin/EDTA solution	GIBCO	Cat# R-001-100
UltraPure distilled water	Invitrogen	Cat# 10977-015
Critical Commercial Assays		
2S Indexed Adaptor Kit	Swift Biosciences	Cat# 26596
Accel-NGS 2S Plus DNA Library Kit	Swift Biosciences	Cat# 21096
EnzCheck Protease assay kit	ThermoFisher Scientific	Cat# E6638
iSCRIPT cDNA synthesis Kit	BIO-RAD	Cat# 178891
High Sensitivity D1000 ScreenTape	Agilent Technologies	Cat# 5067-5584
High Sensitivity D1000 Reagents	Agilent Technologies	Cat# 5067-5585
NEBNext Microbiome DNA Enrichment Kit	New England Biolabs	Cat# E2612
Power SYBR Green Master Mix	Applied Biosystems	Cat# 4367659
PureLink RNA Mini Kit	Invitrogen	Cat# 12183025
PureLink Microbiome DNA Purification Kit	Invitrogen	Cat# A29790
SYBR Green qPCR Master Mix (2X)	Biotool	Cat# B21204
Qubit dsDNA HS assay kit	Invitrogen	Cat# Q32851
Deposited Data		
Metagenomic Sequencing Dataset	This Study	BioProject ID: PRJNA551026
Experimental Models: Cell Lines		
Human Epidermal Keratinocytes, neonatal (HEKn)	GIBCO	Cat# C0015C
Experimental Models: Organisms/Strains		
Mouse: C57BL/6J	Jackson Laboratory	Strain: 000664
Oligonucleotides		
*S. aureus femA* Forward: AACTGTTGGCCACTATGAGT	Paule et al., 2004	N/A
*S. aureus femA* Reverse: CCAGCATTACCTGTAATCTCG	Paule et al., 2004	N/A
*S.epidermidis gseA* Forward: ATGAAAAAGAGATTTTTATCT	Ikeda et al., 2004	N/A
*S.epidermidis gseA* Reverse: GTTTGGTGACACTCTTAAG	Ikeda et al., 2004	N/A
*S. epidermidis ecpA* Forward: TGTGCTTAAAACGCCACGTA	Olson et al., 2014	N/A
*S. epidermidis ecpA* Reverse: GTATAGCCGGCACACCAACT	Olson et al., 2014	N/A
*S. aureus psmα* Forward: TAAGCTTAATCGAACAATTC	Sully et al., 2014	N/A
*S. aureus psmα* Reverse: CCCCTTCAAATAAGATGTTCATATC	Sully et al., 2014	N/A
*S. aureus scpA* Forward: CTATTGCAAACGCTGAGAGC	Mootz et al., 2015	N/A
*S. aureus scpA* Reverse: ACGTACGTCAGTAGGAACACTCTT	Mootz et al., 2015	N/A
*S. aureus sspB* Forward: CAGCAAATTGTTGTTGTGCTAG	Ma et al., 2012	N/A
*S. aureus sspB* Reverse: AAGCCAAAGCCGATTCACACTC	Ma et al., 2012	N/A
TaqMan Murine *Gapdh* primers	Mm99999915_g1 (Applied Biosystems)	N/A
TaqMan Murine *Il6* primers	Mm00446190_m1 (Applied Biosystems)	N/A
TaqMan Murine *Ifng* primers	Mm01168134_m1 (Applied Biosystems)	N/A
TaqMan Murine *Il*4 primers	Mm00445259_m1 (Applied Biosystems)	N/A
TaqMan Murine *Il*17*a* primers	Mm00439618_m1 (Applied Biosystems)	N/A
TaqMan Murine *Il*17*f* primers	Mm00521423_m1 (Applied Biosystems)	N/A
TaqMan Murine *Il1b* primers	Mm00434228_m1 (Applied Biosystems)	N/A
TaqMan Human *GAPDH* primers	Hs02786624_g1 (Applied Biosystems)	N/A
Taqman Human *SPINK5* primers	Hs00928570_m1 (Applied Biosystems)	N/A
Silencer Select *SPINK5* siRNA	s21667 (ThermoFisher)	Cat#4392420
Software and Algorithms		
CLC Assembly Cell (CLC bio, version 3.22.55705)	QIAGEN	https://digitalinsights.qiagen.com/products-overview/analysis-and-visualization/qiagen-clc-assembly-cell/
Clustal Omega (version 1.2.1)	Sievers et al., 2011	http://www.clustal.org/omega/
FragGeneScan (version 1.16)	Kim et al., 2015	https://github.com/hallamlab/FragGeneScanPlus
Geneious R11.1.5	Geneious	https://www.geneious.com
GraphPad Prism (version 5.01)	GraphPad Software	https://www.graphpad.com/
HMMER (version 3.0)	Eddy, 1998	http://hmmer.org/
KneadData, (version 0.5.4)		https://bitbucket.org/biobakery/kneaddata/wiki/Home
metaSPAdes (version 3.11.1)	Nurk et al., 2017	http://cab.spbu.ru/software/meta-spades/
MIDAS (version 1.3.0)	Nayfach et al., 2016	https://github.com/snayfach/MIDAS
PhyloDB (version 1.076)		https://scripps.ucsd.edu/labs/aallen/data/
QIIME 2 (version 2019.1)		https://qiime2.org/
R (version 3.4.1)	R Core team, 2017	https://cran.r-project.org/bin/windows/base/old/3.4.1/

## Highlights

- Netherton subjects have increased *S. aureus* or *S. epidermidis* on their skin
- *S. aureus* PSMα induces greater protease activity in keratinocytes deficient in *SPINK5*
- *S. epidermidis* EcpA protease damages the epidermis
- *S. aureus* and *S. epidermidis* proteases induce skin inflammation in mice
