# Supplementary material for: Interplay of Staphylococcal and Host Proteases Promotes Skin Barrier Disruption in Netherton Syndrome
Source: Cell Rep. Author manuscript; Available in PMC 2020 Apr 25. (PMC7183042; doi:10.1016/j.celrep.2020.02.021)
Supplement: 1 [file NIHMS1571421-supplement-1.pdf]

Cell Reports, Volume *30*

Supplemental Information

Interplay of Staphylococcal and Host Proteases Promotes Skin Barrier Disruption in Netherton Syndrome

Michael R. Williams, Laura Cau, Yichen Wang, Drishti Kaul, James A. Sanford, Livia S. Zaramela, Shadi Khalil, Anna M. Butcher, Karsten Zengler, Alexander R. Horswill, Christopher L. Dupont, Alain Hovnanian, and Richard L. Gallo

A

Subject	Flare	Scaly Erythroderma	ILC	Overall severity
NS1	+	0	3	3
NS2	+	0	3	3
NS3	+	0	1	1
NS4	+	0	3	3
NS5	-	2	0	2
NS6	-	2	0	2
NS7	+	0	3	3
NS8	-	0	3	3
NS9	+	0	3	3
NS10	-	3	0	3

*ILC: Ichthyosis Linearis Circumflexa
*Disease Score: Score: 0=Absent, 1=Minimun, 2=Moderate, 3=Severe, 4=Very Severe

B

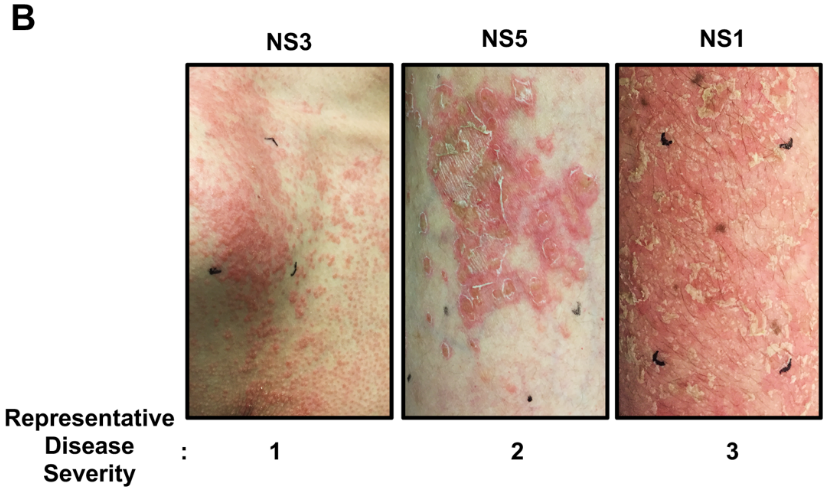

Figure S1. (Related to Figure 1) Netherton syndrome subject disease severity scores.

(A) Table showing representative whole-body severity scores for each subject.

(B) Representative pictures of NS skin with different disease severity (1=minimum, 2=moderate and 3=severe).

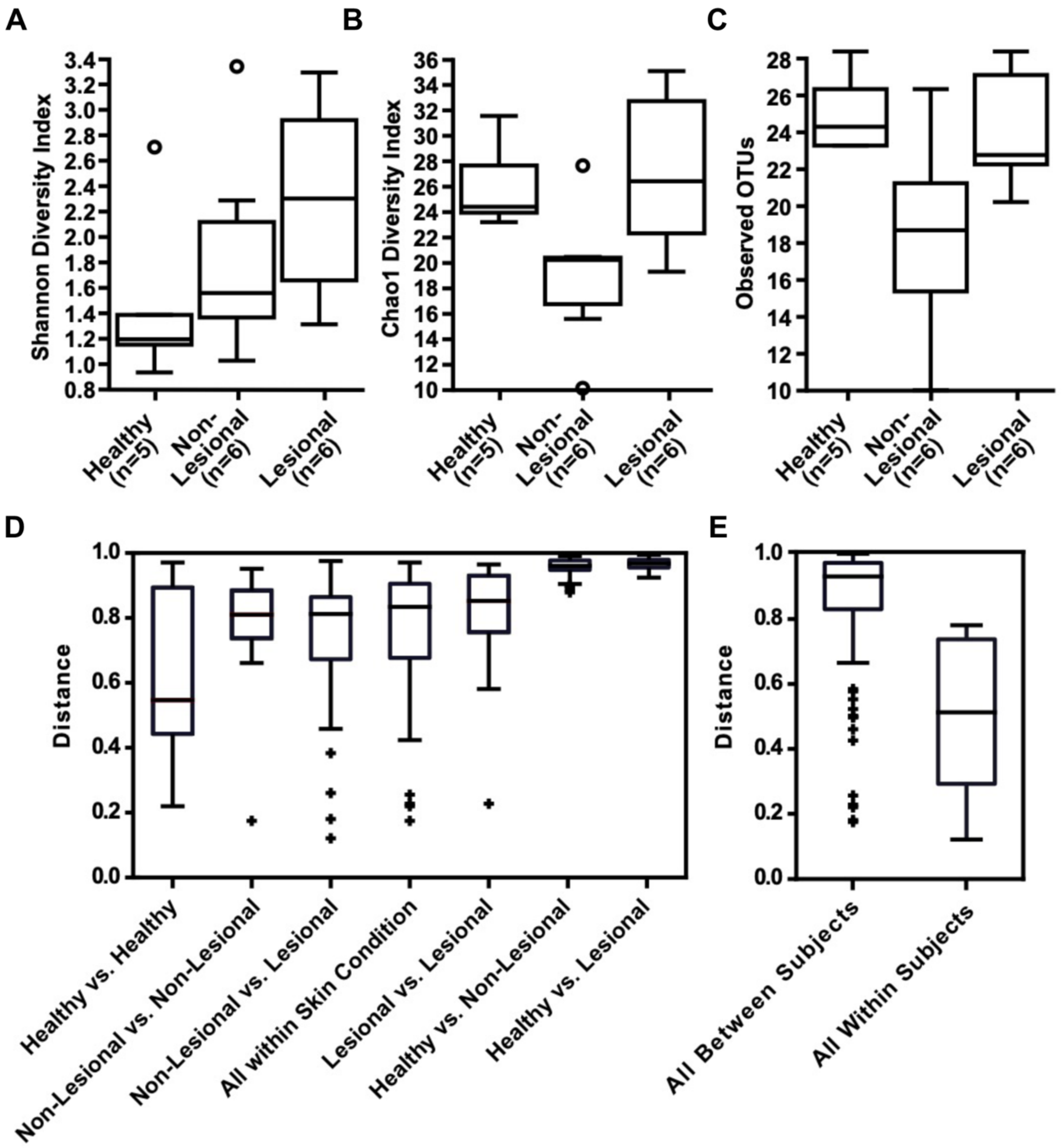

Figure S2. (Related to Figure 1) Netherton syndrome versus healthy control skin bacterial alpha and beta diversity.

(A-C) Alpha diversity measured by **(A)** Shannon diversity index, **(B)** Chao1 diversity index and **(C)** number of observed OTUs for healthy control and NS non-lesional and lesional skin.

(D) Beta diversity distance between and within sample groupings by skin condition.

(E) Beta diversity amongst subjects.

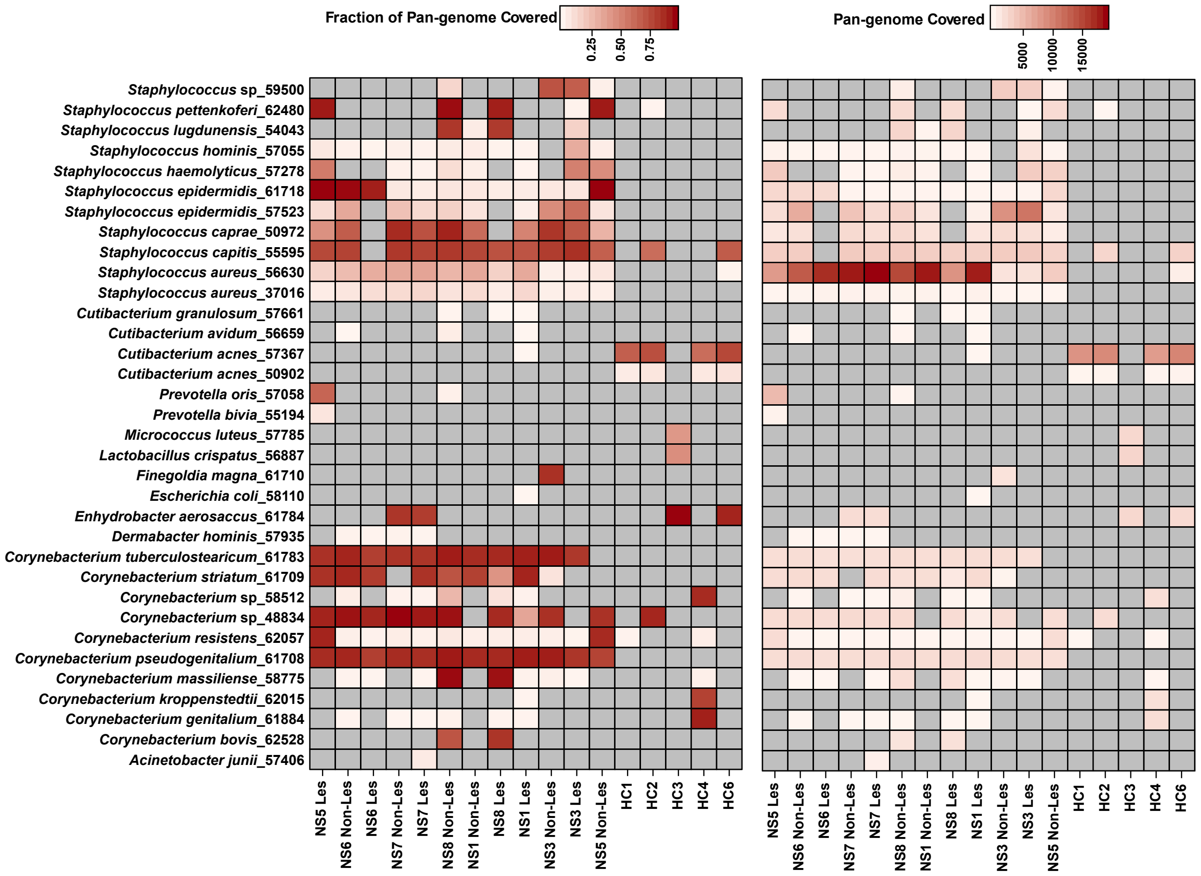

Figure S3. (Related to Figure 1) Pan genome coverage of bacteria species on Netherton skin. Related to Figure 1. Fraction of pan-genome covered across samples, for each prevalent species of interest.

(A) Fraction of pan-genome covered (proportion of non-redundant genes in the reference pan-genome with at least one mapped read).

(B) Number of genes from the pan-genome with at least 1 mapped read.

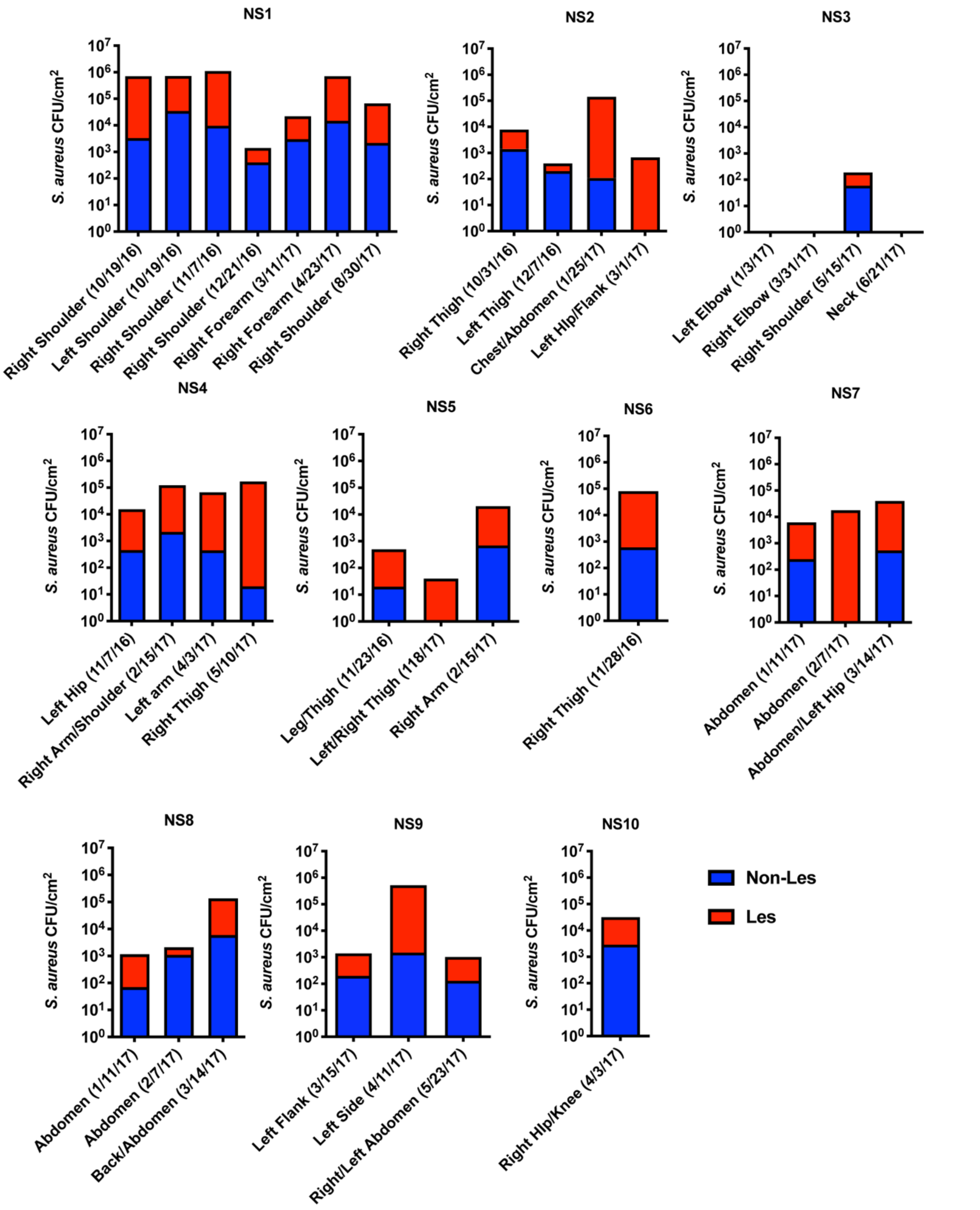

Figure S4. (Related to Figure 2) Live *S. aureus* CFU by individual subject swab site. Comparison of absolute abundance of *S. aureus* per cm^2 of skin swabbed across all Netherton syndrome (NS1-10) subjects with both body site where swabbed and date collected. Both non-lesional (blue bars) and lesional (red bars) were compared as well.

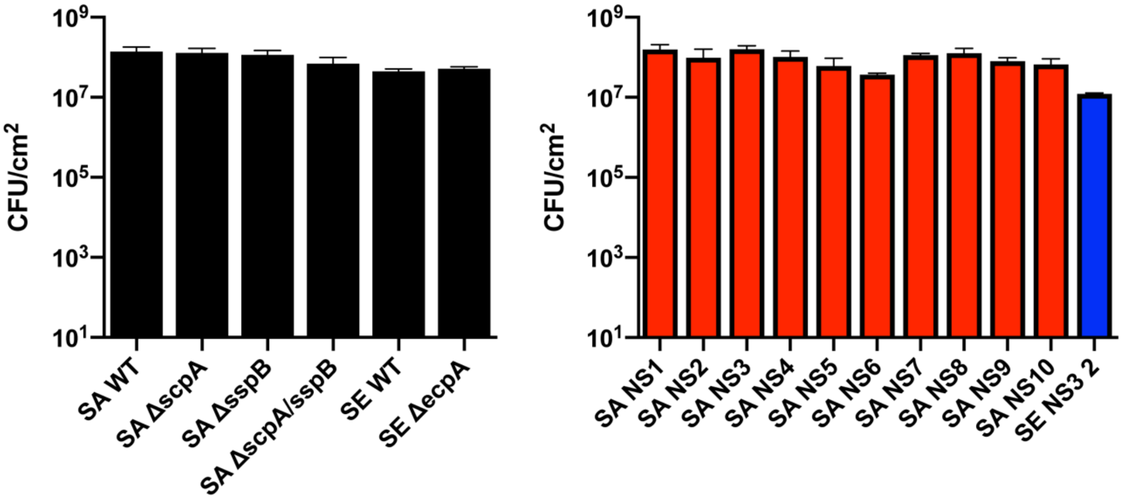

Figure S5. (Related to Figures 3-5) Colony-forming units of bacteria from epicutaneous murine models. Quantity of colony-forming units (CFU) of bacteria per cm^2 of murine back skin after 48h of colonization.

(A) *S. aureus* and *S. epidermidis* cysteine protease knockout bacteria CFU counts.

(B) Clinical staphylococci isolate CFU counts.

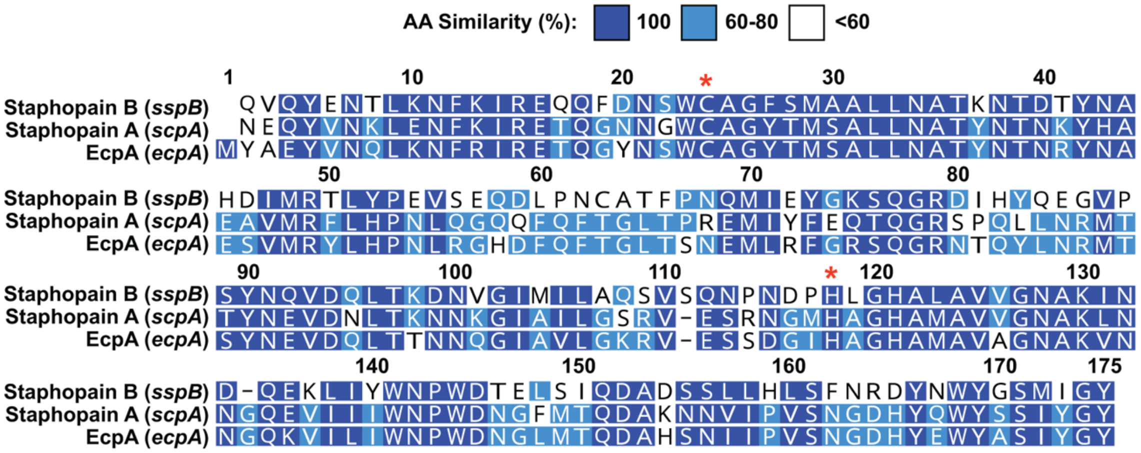

Figure S6. (Related to Figure 5) Comparison of active cysteine protease sequence for Staphopains A/B and EcpA. Alignment of active amino acid sequences for *S. aureus* Staphopain A (*scpA*), Staphopain B (*sspB*), and *S. epidermidis* EcpA (*ecpA*). Asterisks represent bindings sites for cleavage of substrates.

Table S1. (Related to STAR Methods) Netherton syndrome subjects involved in this study.

Subject	Sex	Age (Years)	Sampling date	Swab site
NS1	F	14	10/19/2016	Right and Left Shoulder[M]
		14	11/7/2016	Right Shoulder
		15	12/21/2016	Right Shoulder
		15	3/11/2017	Right Shoulder
		15	4/23/2017	Right Arm and Right Forearm
		15	6/12/2017	Left Leg
		15	8/30/2017	Right Shoulder
NS2	M	12	10/31/2016	Right Thigh
		12	12/7/2016	Left Thigh
		12	1/25/2017	Chest (NL) Abdomen (L)
		12	3/1/2017	Flank R&L (NL) Hip R&L (L)
NS3	M	16	3/1/2017	Right and Left Elbow[M]
		16	3/31/2017	Right Elbow
		16	5/15/2017	Right Shoulder
		17	6/21/2017	Neck
NS4	F	27	11/7/2016	Left Hip
		28	2/15/2017	Right Arm (NL) Right Shoulder (L)
		28	4/3/2017	Left Arm
		28	5/10/2017	Right and Left Thigh
NS5	F	35	11/23/2016	Leg and Thigh[M]
		35	1/18/2017	Left Thigh (NL) Right Thigh (L)
		35	2/15/2017	Left and Right Arm
NS6	M	42	11/28/2016	Right Thigh[M]
NS7	F	45	1/11/2017	Abdomen[M]
		45	2/7/2017	Abdomen
		45	3/14/2017	Abdomen (NL) Left Hip (L)
NS8	F	47	1/11/2017	Abdomen[M]
		47	2/7/2017	Abdomen
		47	3/14/2017	Back (NL) Abdomen (L)
NS9	F	18	3/15/2017	Left Flank
		18	4/11/2017	Left Flank
		18	5/22/2017	Right Abdomen
NS10	M	28	4/3/2017	Right Hip Right Knee Right Thigh

***NS=Netherton syndrome; NL=Non-lesional; L=Lesional**
***M=Samples used from metagenomic analysis**

Table S2. (Related to STAR Methods) Healthy controls involved in this study.

Subject	Sex	Age (Years)	Sampling date	Swab Site
HC1	F	28	1/2/2018	Arm[M]
			1/2/2018	Abdomen
			1/2/2018	Leg
HC2	F	38	2/1/2018	Arm[M]
			2/1/2018	Abdomen
			2/1/2018	Leg
HC3	F	27	2/1/2018	Arm
			2/1/2018	Abdomen
			2/1/2018	Leg[M]
HC4	F	27	2/1/2018	Arm
			2/1/2018	Abdomen
			2/1/2018	Leg[M]
HC5	M	30	2/1/2018	Arm
			2/1/2018	Abdomen
			2/1/2018	Leg
HC6	M	33	2/1/2018	Arm
			2/1/2018	Abdomen[M]
			2/1/2018	Leg
HC7	F	52	2/1/2018	Arm
			2/1/2018	Abdomen
			2/1/2018	Leg
HC8	F	38	2/1/2018	Arm
			2/1/2018	Abdomen
			2/1/2018	Leg

***HC=Healthy control subject**
***M=Samples used from metagenomic analysis**

Table S3. (Related to STAR Methods) Metagenomic library prep and sequencer read count data.

Samples	Sequencing Code Names	I7 INDEX ID (SWIFT A+B)	INDEX	I5 INDEX ID	INDEX 2	Peak Size (Average)	Total Reads	Trimmed Reads (% of Total Reads)	Human Reads	Non-Human Reads (%)
HC1	HC1	A002	CGAT GTAT	Univ	TCTTT CCC	475	1.49E+06	8.22E+05 (55.19)	4.44E+05	3.78E+05 (45.96)
HC2	HC2	A004	TGAC CAAT	Univ	TCTTT CCC	489	2.63E+06	1.16E+06 (44.22)	2.68E+05	8.96E+05 (76.99)
HC3	HC3	A013	AGTC AACA	Univ	TCTTT CCC	454	3.57E+06	1.74E+06 (48.83)	5.99E+05	1.14E+06 (65.59)
HC4	HC4	A014	AGTTC CGT	Univ	TCTTT CCC	471	2.58E+06	1437158 (55.68)	6.07E+05	8.30E+05 (57.77)
HC6	HC6	A015	ATGTC AGA	Univ	TCTTT CCC	446	1.82E+06	9.67E+05 (53.24)	1.94E+05	7.72E+05 (79.89)
NS1 Non-Lesional	1M	A016	CCGTC CCG	Univ	TCTTT CCC	461	3.38E+07	1.80E+07 (53.36)	1.53E+07	2.68E+07 (14.89)
NS1 Lesional	2M	A018	GTCC GCAC	Univ	TCTTT CCC	469	2.35E+07	1.24E+07 (52.81)	9.28E+06	3.14E+06 (25.30)
NS5 Non-Lesional	9M	A019	GTGA AACG	Univ	TCTTT CCC	457	2.31E+06	1.26E+06 (54.47)	1.52E+05	1.10E+06 (87.88)
NS5 Lesional	10M	A001	ATCA CGAT	Univ	TCTTT CCC	474	5.51E+06	3.20E+06 (58.14)	1.79E+06	1.41E+06 (44.06)
NS6 Non-Lesional	11M	A003	TTAG GCAT	Univ	TCTTT CCC	480	1.39E+07	5.95E+06 (42.67)	5.04E+06	8.90E+05 (15.00)
NS6 Lesional	12M	A008	ACTTG AAT	Univ	TCTTT CCC	469	3.27E+07	1.66E+07 (50.81)	1.50E+07	1.63E+06 (9.81)

Samples	Sequencing Code Names	I7 INDEX ID (SWIFT A+B)	INDEX	I5 INDEX ID	INDEX 2	Peak Size (Average)	Total Reads	Trimmed Reads (% of Total Reads)	Human Reads	Non-Human Reads (%)
NS7 Non-Lesional	17M	A009	GATC AGAT	Univ	TCTTT CCC	460	1.83E+07	1.03E+07 (56.55)	8.05E+06	2.27E+06 (21.97)
NS7 Lesional	18M	A020	GTGG CCTT	Univ	TCTTT CCC	427	1.13E+07	5.94E+06 (52.34)	3.90E+06	2.04E+06 (34.33)
NS8 Non-Lesional	19M	A021	GTTTC GGA	Univ	TCTTT CCC	424	5.17E+07	2.85E+07 (55.11)	2.32E+07	5.42E+06 (18.66)
NS8 Lesional	20M	A022	CGTA CGTA	Univ	TCTTT CCC	423	4.32E+07	2.33E+07 (53.84)	2.08E+07	2.46E+06 (10.59)
NS3 Non-Lesional	35M	A023	GAGT GGAT	Univ	TCTTT CCC	424	3.37E+06	1.56E+06 (46.14)	7.28E+05	8.29E+05 (53.25)
NS3 Lesional	36M	A025	ACTG ATAT	Univ	TCTTT CCC	413	4.02E+06	2.13E+06 (52.90)	1.40E+05	1.99E+06 (93.41)
